# Influence of different knee and ankle ranges of motion on the elasticity of triceps surae muscles, Achilles tendon, and plantar fascia

**DOI:** 10.1038/s41598-020-63730-0

**Published:** 2020-04-20

**Authors:** Chun-Long Liu, Ji-Ping Zhou, Peng-Tao Sun, Bai-Zhen Chen, Jun Zhang, Chun-Zhi Tang, Zhi-Jie Zhang

**Affiliations:** 1 Clinical Medical College of Acupuncture, Moxibustion and Rehabilitation, Guangzhou University of Chinese Medicine, Guangzhou, China; 2 Department of Medical Ultrasound, The Second Affiliated Hospital of Guangzhou University of Chinese Medicine, Guangzhou, China; 3 Luoyang Orthopedics Hospital of Henan Province, Luoyang, China; 4 Nan’ao people’s Hospital, Dapeng New District, Shenzhen, China

**Keywords:** Whole body imaging, Predictive markers

## Abstract

Stiffness is a valuable indicator of the functional capabilities of muscle-tendon-fascia. Twenty healthy subjects participated in this study in which the passive elastic properties of the medial gastrocnemius (MG), lateral gastrocnemius (LG), soleus muscles (SOL), Achilles tendon (AT, at 0 cm, 3 cm and 6 cm proximal to the calcaneus tubercle, corresponding to AT0cm, AT3cm and AT6cm, respectively) and plantar fascia (PF) were quantified when their knee was fully extended or flexed to 90° using shear wave elastography at 25° of dorsiflexion (DF25°), 0° (neutral position) of flexion, and 50° of plantar flexion (PF50°) of the ankle joint. The stiffnesses of the AT, MG, LG, SOL and the fascia with the knee fully extended were significantly higher than those with the knee flexed to 90° (p < 0.05), while the stiffness of the PF showed the opposite relationship (p < 0.05). When the knee was fully extended, the stiffness was higher in the LG than in the MG at PF50° and 0° (p < 0.01), and it was higher in the MG than in the LG at DF25° (p = 0.009). Nevertheless, regardless of the knee angle, the stiffness decreased from AT3cm > AT0cm > AT6cm at PF50° and 0° (p < 0.001), while the stiffness decreased from AT0cm > AT3cm > AT6cm at DF25°. Regardless of the knee and ankle angles, the stiffness of the PF increased in a proximal-to-distal direction (p < 0.001). These insights can be used to gain a more intuitive understanding of the relationships between the elastic properties of the muscle-tendon unit and its function.

## Introduction

Achilles tendon (AT) injuries and plantar fasciitis can cause chronic pain and impairment^1^. In the clinic, the type of treatment administered depends in part on the region and intensity of pain. For example, eccentric exercises are more effective in the treatment of mid-substance tendinopathy than in the treatment of insertional tendinopathy^1^. However, because of the complexity of the anatomy and mechanics of the AT and plantar fascia (PF), it can be challenging to identify the region and severity of the damage. In terms of anatomy, the AT, which is the conjoined tendon of the medial gastrocnemius (MG), the lateral gastrocnemius (LG), and the soleus (SOL), is an important bridge for walking, running and jumping in daily life, and each of the contributing muscles exhibit unique structural features^2,3^. The twisted structure of the AT is formed by the MG tendons, LG tendons and SOL tendons. These muscle tendons rotate as they descend but do not run parallel to each other^4^. In addition, the superficial layer of the AT is formed by the MG tendons, while the deep layer consists of the LG tendons and SOL tendons^5^. Furthermore, the LG is shorter and smaller than the MG, so the MG and LG differ in terms of muscle strength and their contribution to the AT^6^. In other words, these regional structural features (including those of the MG, LG, SOL, AT and PF) have great inherent variability, which may affect the position and severity of damage in an individual^6^. Therefore, it is necessary to further understand the relationship between muscle-tendon-fascia anatomy and mechanical properties. This knowledge may greatly improve our ability to distinguish normal regional changes in muscle-tendon-fascia biomechanical properties from those arising due to damage.

The passive mechanical properties of muscle/tendon/fascia play an important role in the completion of each movement. It has been reported that the incidence rate of Achilles tendinitis is 2.35‰ in the general population^7^, while the lifetime prevalence of plantar fasciitis is approximately 10% in the general population^8^. Pain caused by Achilles tendinitis and plantar fasciitis can reduce the use intensity of the affected foot, decrease the flexibility of muscle/tendon, and ultimately, lead to muscle/tendon contractures^1,9–11^. Muscle/tendon contractures are the most obvious symptoms and are characterized by decreased muscle lengths/tendon lengths and increased passive stiffness values. They can lead to reduced joint ranges of motion (ROM) during walking and running^12^. Traditional metrics used to assess passive mechanical properties include the passive ROM and the global joint torques generated by motion resistance. However, the passive ROM cannot be used to distinguish the different structures crossing the joint, such as the skin, joint capsule, synergistic muscles, tendons, nerves and fasciae. Therefore, an investigation of the intermuscle and intratendon/PF distribution of passive stiffness *in vivo* would provide more information on the muscle-tendon-fascia biomechanics, allowing clinicians to optimize treatment strategies by, for example, targeting stiffer locations.

Stiffness, one of the properties of soft tissue, is defined as the ability to resist external forces or deformations from an initial shape^13^. The passive elastic forces of muscles and tendons and stretch forces determine the stiffness of individual muscle-tendon-fascia units. Stiffness is one of the most difficult indicators to quantify, although it is a commonly used parameter in clinical examinations of muscles and tendons. In addition, soft tissue is dynamic, so the stiffness also changes. For instance, the stiffness of muscles can change during contractions, while that of passive tissues such as tendons can change due to strain stiffening. Thus, accurate assessments of the stiffness of soft tissues across their entire functional range are essential for determining the normal stiffness level and improving their physiological function.

Currently, magnetic resonance elastography (MRE) and MyotonPRO devices are used to quantitatively assess the stiffness of soft tissues. Green *et al*.^14^ used MRE to examine the anisotropic properties of muscles, and Orner *et al*.^15^ used MyotonPRO to quantify AT stiffness. However, MRE is not frequently used to quantify stiffness because the equipment is expensive and not portable, and whether there is an effect of tight skin on MyotonPRO measurements has not yet been determined. Shear wave elastography (SWE) is a noninvasive technique used to assess the stiffness of soft tissues. It can be used to quantify the stiffness of a regional area using its relationship with shear wave speed: E = 3ρv^2^ (E: shear modulus, ρ: tissue density, v- shear wave velocity)^16^. Many studies have reported the use of SWE to assess the stiffness of skeletal muscles, tendons and PF. For example, our previous studies have shown that SWE is a valid and reliable tool for quantifying the stiffness of the gastrocnemius and AT. The intraclass correlation coefficient was 0.74 to 0.95^17^. In addition, Eby *et al*. also demonstrated a positive significant correlation between muscle stiffness measurements obtained by SWE and stiffness measurements obtained by a material testing system^18^. Furthermore, SWE was used not only to quantify the stiffness of physiological soft tissues, such as the gastrocnemius, patellar tendon, PF and AT, but also to quantify the stiffness of pathological soft tissues, such as the muscles of patients with spastic cerebral palsy or poststroke spasms and the PF of patients with plantar fasciitis^19–23^. In summary, SWE can be used to monitor variations in the stiffness of muscle-tendon-fascia units at different knee and ankle joint angles.

The aim of this study was to characterize the effects of different ankle joint angles with the knee fully extended or flexed to 90° on the stiffness of the MG, LG, SOL, AT and PF.

## Materials and methods

### Ethics statement

The present study protocol was approved by the Human Subjects Ethics Committee of Luoyang Orthopedics Hospital of Henan Province (KY2019-001-01) and conformed to the Declaration of Helsinki. Before participation, all participants were fully informed of the aim and experimental procedures of the study and signed the informed consent form.

### Participants

Twenty healthy males were recruited in this study. The mean age, height, and body mass were 21.3 ± 0.86 (19–23) years, 171.25 ± 4.99 (162–181) cm, and 61.02 ± 5.6 (50–75) kg, respectively. The inclusion criteria were that all participants were healthy and did not have a history of ongoing neuromuscular diseases or musculoskeletal injuries specific to the ankle or knee joints over the previous six months. The exclusion criteria included pain in the PF or AT and an anomaly on ultrasound.

### Equipment and parameter settings

An ultrasound SWE system (Aixplorer Supersonic Imagine, FRANCE) with a 40-mm linear array transducer (SL15-4, Supersonic Imagine, FRANCE) was used in this study. The settings of the SWE system were set as follows: the instrument was set to the musculoskeletal mode. The frequency was 4~15 MHz. The SWE mode was the penetration mode. The opacity was 85%. The elastic modulus range for the muscles and AT was 0~800 kPa, while the elastic modulus range for the PF was 0~300 kPa. The depth of the B-scan was 3.0 cm. For the AT, the Q-box diameter was defined by the thickness of the tendon, which was the distance between the superior and inferior borders of the AT^17^. For the MG, LG and SOL, the size of the regions of interest (ROIs) had to be set to 10*10 mm, and the diameters of the Q-box of muscle and muscle fascia were 5 mm and 1 mm, respectively^17^. For the PF, the three quantification areas were located between the calcaneal insertion of the PF and 1 cm distal to its insertion, and the Q-box diameter was determined by the thickness of the fascia, which was defined as the distance between the superior and inferior borders of the PF^19–21,23^.

### Experimental design and protocol

The dominant leg was identified by asking the participant to kick a ball (kicking preference)^24^. Participants were asked to wear loose-fitting clothes. During testing, each participant was instructed to lay in aprone-lying position with their feet relaxed and hanging over the lower edge of the treatment bed, the hip and knee joints fully extended, and the upper limbs placed alongside the body^24^. The ankle joint was fixed using a customized and movable ankle foot orthosis at a designated angle^25^. The measurement sites for the AT were 0 cm (AT0cm), 3 cm (AT3cm) and 6 cm (AT6cm) proximal to the calcaneal tuberosity^26^, while those for the MG and LG were defined as the proximal 30% of the lower leg length^17^. The SOL was measured in the distal half of the SOL for visualization of the distal SOL fascicles alone^27^. The PF was assessed at 3 successive levels: between the first and second metatarsal bone, on the anterior edge of the inferior calcaneal border and in the posterior section of the PF^8,28^. The length was measured with a tape measure, and a black pen was used to mark the location of the measurement site. The stiffness was measured with the SWE transducer positioned on the marks on the skin for the MG, LG, SOL, AT and PF with the ankle joint positioned at 25° of dorsiflexion (DF25°), in a neutral position (0°), or at 50° of plantar flexion (PF50°) and the knee fully extended or flexed to 90°. In addition, to reduce experimental errors, the participants were explicitly asked to keep their lower extremities fully relaxed throughout testing and to refrain from high-intensity exercise for 48 hours before testing^17,28^.

### Shear wave ultrasound elastography

**Figure 1:**
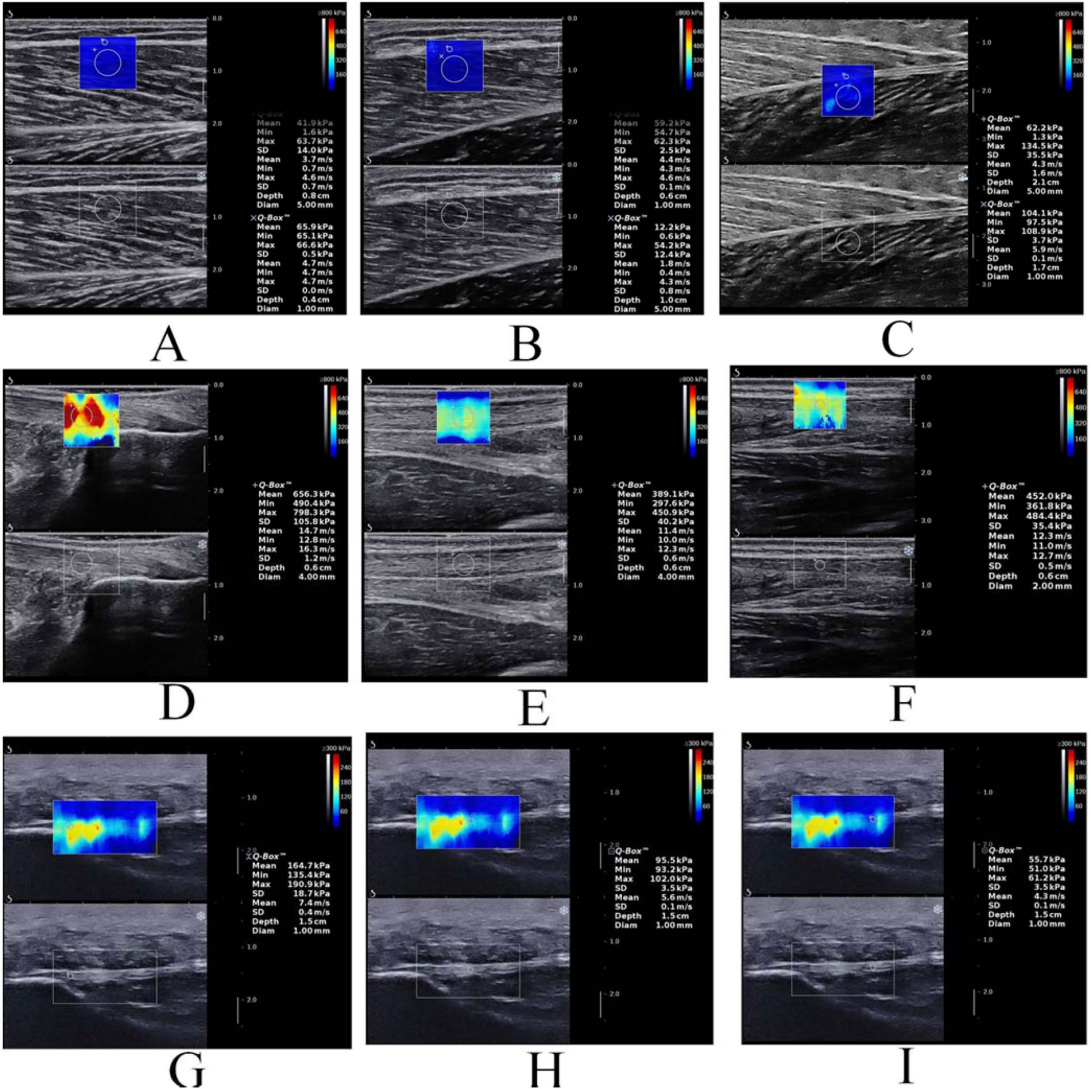
A longitudinal SWE sonogram of the MG, LG, SOL, AT and PF showing the measurements of the shear modulus at different regions (from A to I). The color scale for the muscle-tendon-fascia elasticity is shown in the upper images. The longitudinal grayscale sonograms of the muscle-tendon-fascia are shown in the bottom images. The Q-Box is shown on the right. (**A**) The shear modulus values of the LG; (**B**) the shear modulus values of the MG; (**C**) the shear modulus values of the SOL; (**D–F**) the shear modulus values of AT0cm, AT3cm and AT6cm, respectively; (**G–I**) the shear modulus values of PF-pro, PF-mid and PF-dis, respectively.

Before the measurements, the participants were asked to relax for 5 min to ensure that the triceps surae was relaxed. During testing, sufficient ultrasound gel was applied between the skin and the transducer to facilitate the transmission of ultrasound waves. The midpoint of the transducer was placed perpendicular to the skin’s surface, where we had previously made a mark, with light pressure. Then, SWE was initiated to examine the shear modulus of the muscle (with clear visibility of the muscle fascicles and aponeuroses) or the tendon/PF (with clear visibility of the superficial layer and deep layer and the surrounding tissue)^8,29^ (Fig. 1). During the acquisition of the SWE images, the transducer was maintained motionless for at least 5–8 s^17^. Then, the grayscale image showed the longitudinal section of the muscle or tendon. Image quality was closely monitored while the measurements were made. When the color in the ROIs was uniform and several fibers were continuously visible, the images were frozen. The mean value of the shear modulus (kPa) was obtained from the system and stored for off-line analysis. Each angle was measured at 1-min intervals. Three measurements were performed for each location. The mean of the elastic moduli from all 3 images was used for further analyses.

### Statistical analysis

Statistical analysis was performed using SPSS Version 19.0 (SPSS, Chicago, IL). All data are presented as the mean ± standard deviation. The Kolmogorov-Smirnov (KS) test was used to determine whether all stiffness data had a normal distribution. The distributions consistently passed the KS test. For the stiffness data, separate 3-way analyses of variance (ANOVAs) (knee angle×ankle angle×muscle/tendon/fascia) with repeated measures were performed. If there was a knee angle×ankle angle×muscle/tendon/fascia interaction, the main effects were investigated, and a Bonferroni correction was performed. One-way repeated measures ANOVA with Bonferroni’s post hoc test was used to determine the differences in stiffness between the ankle positions or between muscle/tendon/fascia units. In addition, the effect size was also calculated using Cohen’s d^30^. Cohen’s d values less than 0.2, 0.2–0.5, and greater than 0.8 correspond to small, medium, and large effects, respectively. The statistical significance level was set to be p < 0.05 (α = 0.05).

## Results

### Effect of knee angle on stiffness

The relationships between muscle-tendon-fascia stiffness and passive knee angle are shown in Tables 1 and 2. The stiffness of the AT, MG, LG, SOL and the fascia with the knee fully extended was significantly higher than that with the knee flexed to 90° (p < 0.05). However, when the ankle was passively dorsiflexed to 0° and 25°, the stiffness of the PF with the knee fully extended was less than that with the knee flexed to 90° (p < 0.05). There was no significant effect of knee angle on PF stiffness when the ankle was positioned at PF50° (p > 0.05).

**Table 1:** Effect of ankle or knee angles on the stiffness of muscle and tendon.

Measurement position		Ankle angle	Knee 0°	Knee 90°	Cohen’s d	P value
MG		PF50°	20.58 ± 7.10	12.02 ± 3.89	1.495	0.000
		0°	46.78 ± 8.51	13.23 ± 3.26	5.206	0.000
		DF25°	121.56 ± 28.8	19.26 ± 4.90	4.952	0.000
LG		PF50°	26.91 ± 7.89	10.25 ± 3.64	2.712	0.000
		0°	53.70 ± 13.87	15.36 ± 6.95	3.495	0.000
		DF25°	103.89 ± 25.66	34.17 ± 10.82	3.541	0.000
SOL		PF50°	74.41 ± 29.29	41.93 ± 11.98	1.452	0.000
		0°	141.15 ± 43.43	66.03 ± 17.62	2.267	0.000
		DF25°	132.00 ± 35.01	111.94 ± 26.69	0.644	0.035
AT	0 cm	PF50°	356.59 ± 54.99	276.22 ± 44.33	1.609	0.000
		0°	583.66 ± 64.63	528.31 ± 74.61	0.793	0.025
		DF25°	646.49 ± 72.67	601.56 ± 50.49	0.718	0.027
	3 cm	PF50°	389.30 ± 66.41	323.06 ± 56.54	1.074	0.001
		0°	604.35 ± 66.40	552.32 ± 92.17	0.648	0.028
		DF25°	622.97 ± 48.68	577.72 ± 46.83	0.947	0.011
	6 cm	PF50°	363.23 ± 58.57	295.70 ± 46.32	1.279	0.000
		0°	566.43 ± 74.09	476.20 ± 105.02	0.993	0.006
		DF25°	601.06 ± 44.18	541.61 ± 56.70	1.170	0.003

AT: Achilles tendon, MG: Medial head of the gastrocnemius muscle, LG: Lateral head of the gastrocnemius muscle.

PF50°: plantar flexion 50°, 0°: neutral position, DF25°: dorsiflexion 25°.

**Table 2:** Effect of ankle or knee angle on the stiffness of fascia.

Measurement position		Ankle angle	Knee 0°	Knee 90°	Cohen’s d	P value
MG Fascia		PF50°	31.07 ± 9.17	11.50 ± 4.61	2.697	0.000
		0°	67.31 ± 18.89	13.90 ± 3.06	3.947	0.000
		DF25°	194.43 ± 54.45	23.10 ± 8.19	4.400	0.000
LG Fascia		PF50°	42.86 ± 12.37	9.83 ± 3.63	3.623	0.000
		0°	84.47 ± 18.80	18.79 ± 11.63	4.202	0.000
		DF25°	177.01 ± 46.03	38.34 ± 14.57	4.062	0.000
SOL Fascia		PF50°	104.25 ± 33.61	50.53 ± 13.42	2.099	0.000
		0°	224.09 ± 67.27	114.53 ± 25.73	2.151	0.000
		DF25°	261.12 ± 46.70	219.54 ± 55.74	0.809	0.012
PF	Pro	PF50°	52.51 ± 26.80	56.55 ± 17.75	0.178	**0.551**
		0°	66.98 ± 23.74	89.79 ± 34.21	0.775	0.016
		DF25°	78.37 ± 26.58	103.23 ± 39.07	0.744	0.014
	Mid	PF50°	44.66 ± 25.59	48.54 ± 14.15	0.188	**0.460**
		0°	52.57 ± 20.22	68.07 ± 28.66	0.625	0.042
		DF25°	54.48 ± 20.57	67.93 ± 24.98	0.588	0.036
	Dis	PF50°	33.66 ± 20.35	38.00 ± 13.23	0.253	**0.325**
		0°	35.88 ± 11.99	53.98 ± 24.44	0.940	0.003
		DF25°	38.43 ± 13.52	54.26 ± 18.66	0.972	0.000

MG: Medial head of the gastrocnemius muscle, LG: Lateral head of the gastrocnemius muscle, PF: plantar fascia.

PF50°: plantar flexion 50°; 0°: neutral position; DF25°: dorsiflexion 25°.

Pro: proximal; Mid: Middle; Dis: Distal.

### Differences in stiffness among muscles, tendon and fascia

**Figure 2:**
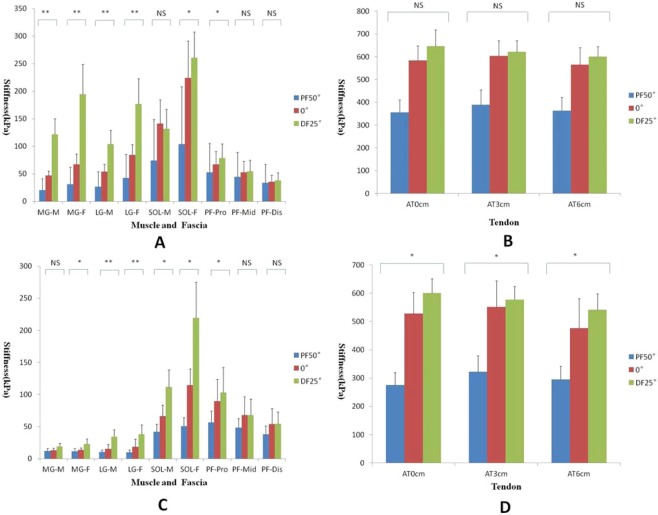
Variations in the stiffness of the MG, MG fascia, LG, LG fascia, SOL, SOL fascia and PF at –50° (blue), 0° (red), and 25° (green) of ankle joint flexion with the knee fully extended/flexed to 90°. (**A**,**B**) The knee fully extended, (**C**,**D**) the knee flexed to 90°. *Comparisons were carried out between any two groups, and all P values were less than 0.05. **Comparisons were carried out between any two groups, and all P values were less than 0.001. NS (nonsignificant) Comparisons were carried out between any two groups, and all P values were greater than 0.05. M: muscle, F: fascia.

**Figure 3:**
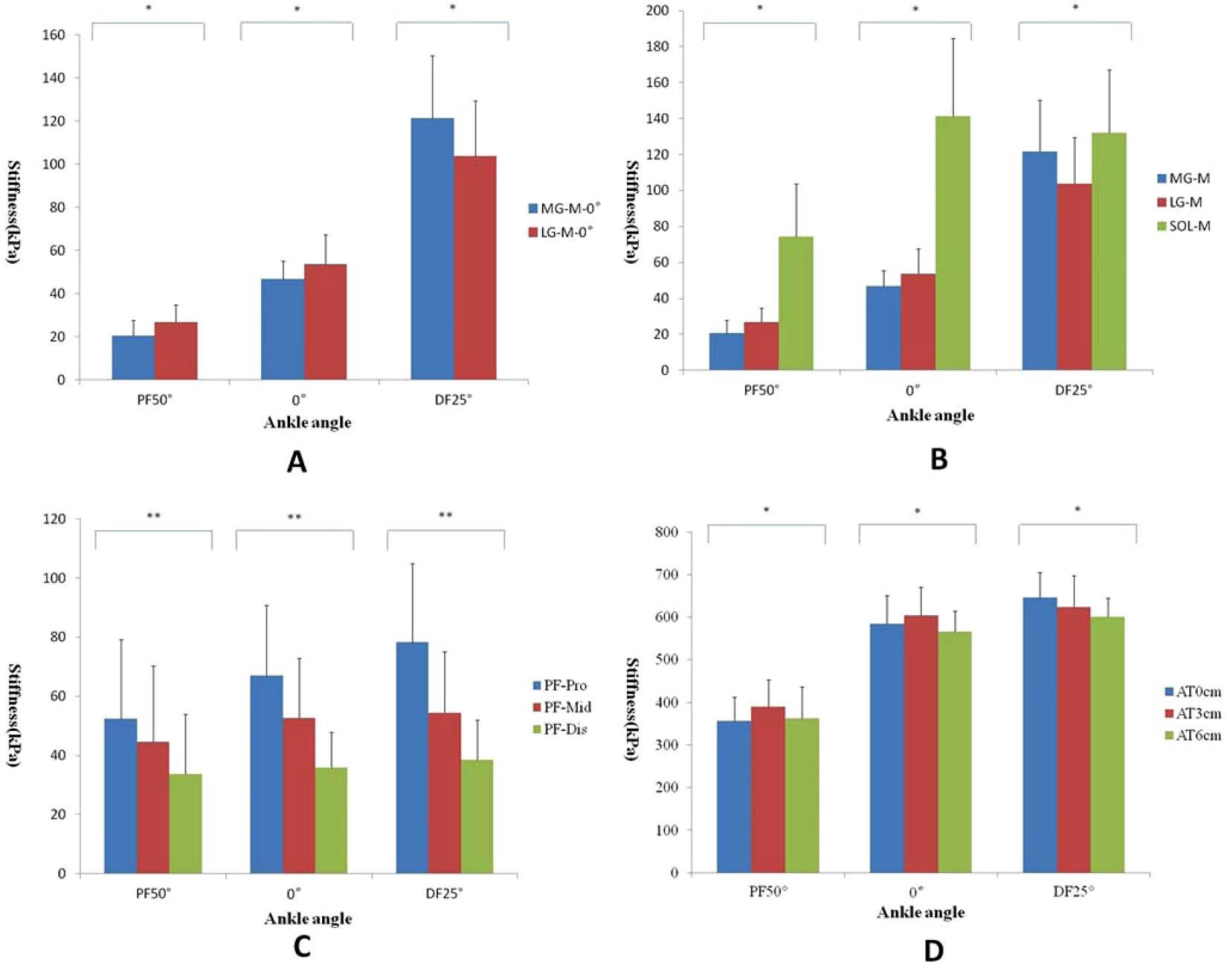
Variations in the stiffness of the MG, LG, SOL, AT and PF at –50°, 0°, and 25° of ankle joint flexion with the knee fully extended. *Comparisons were carried out between any two groups, and all P values were less than 0.05. **Comparisons were carried out between any two groups, and all P values were less than 0.001.

The relationships between stiffness and passive ankle angles are shown in Figs. 2 and 3. There were significant differences among the 3 ankle angles in terms of the stiffness of the MG, MG fascia, LG, LG fascia, SOL fascia and PF-proximal (PF-pro) with the knee fully extended, and stiffness increased with increasing ankle dorsiflexion (p < 0.05). There were no significant differences in the stiffness of the AT, PF-mid, PF-dis or SOL (p > 0.05).

There were significant differences among the 3 ankle joint angles in terms of the stiffness of the SOL, MG fascia, LG, LG fascia, SOL fascia, and PF-pro with the knee flexed to 90° (p < 0.05), and no significant difference was found in the stiffness of the MG, PF-mid, or PF-dis. For the 3 regions of the AT, the stiffness increased with increasing ankle dorsiflexion (p < 0.05).

Additional analysis showed a significant difference between the MG and LG with the knee fully extended. The stiffness was higher in the LG than in the MG at PF50° and 0° (p < 0.01) and in the MG than in the LG at DF25° (p = 0.009). However, no significant difference was found when the knee was flexed to 90°. For the three different regions of the AT, regardless of the knee angle, the stiffness decreased from AT3cm to AT0cm to AT6cm at PF50° and 0° (p < 0.001), while the stiffness decreased from AT0cm to AT3cm to AT6cm at DF25°. For the 3 regions of the PF, regardless of the knee and ankle angles, the stiffness increased in a proximal-to-distal direction (p < 0.001).

## Discussion

The main findings of the present study were that 1) the stiffness of the AT, MG, LG, SOL and fascia with the knee fully extended was significantly higher than that with the knee flexed to 90°, while the stiffness of the PF showed the opposite relationship. 2) When the knee was flexed to 90°, a significant increase in PF stiffness was observed at 0° and DF25°. 3) When the knee was fully extended, the stiffness was higher in the LG than in the MG at PF50° and higher in the MG than in the LG at DF25°. 4) Regardless of the knee position, the stiffness decreased from AT3cm to AT0cm to AT6cm at PF50° and 0°, while the stiffness decreased from AT0cm to AT3cm to AT6cm at DF25°. 5) For the 3 regions of the PF, regardless of the knee and ankle positions, the stiffness increased in a proximal-to-distal direction.

### Effects of knee angle on muscle/tendon/PF stiffness

The results of this study showed high variability in the passive stiffness of muscles, tendons and fascia when the knee was fully extended or flexed at 90°. Compared to the stiffness when the knee was flexed to 90°, the stiffness of the AT, MG, LG, SOL and fascia with the knee fully extended were significantly higher. Our results are consistent with those recently reported^31–35^. For example, Hug *et al*. found that the shear modulus of the MG and AT with the knee fully extended is larger than that with the knee flexed to 90°^32^, and Le *et al*. found that the shear modulus of the MG, LG and SOL with the knee fully extended is larger than that with the knee flexed at 90°^33^. When sufficient mechanical stress is imposed on the lower leg, passive movement of the joint can significantly change the stiffness of the muscle-tendon-fascia unit^4,35^. The innovative aspect of the present study is that a quantitative analysis of different positions of the muscle fascia and PF. According to our findings, muscle-tendon-fascia units exhibit different stiffness changes at different angles of the knee joint. In addition, we found that when the ankle was passively dorsiflexed from 0° to 25°, the stiffness of the PF with the knee fully extended was less than that with the knee flexed to 90° and that there was no difference at PF50°. A possible explanation for this result is that when the knee is flexed to 90°, passively dorsiflexing the ankle affects the PF, while the force is shared by the PF and AT when the knee is fully extended. Therefore, we suggest that individuals fully extend their knee when they want to fully stretch their calf muscles and AT and to flex the knee when they want to fully stretch the PF. In addition, the changes in the synergistic muscles, internal tendons and internal fascia associated with different stiffnesses in the lower leg between the experiments (i.e., with the knee fully extended or flexed to 90°) may be associated with the anatomical configuration of the muscles, tendons and fascia (i.e., the biarticular structure of the gastrocnemii, the mono-articular structure of the SOL, and the different compositions and structures across regions of the AT/PF) and their passive force-length relationships^4,6,27,31^.

### Effects of the ankle angle on muscle/tendon/PF stiffness

We found that the stiffness of muscles, ATs and PF increased with increasing ankle dorsiflexion. Our findings concerning the stiffness estimation using SWE are in agreement with those reported in the studies of Hug *et al*.^32^ and Le *et al*.^33^. These authors found that the shear moduli of muscles and tendons increase with passive ankle dorsiflexion. Cheng *et al*. found that the strain of PF increases as the dorsiflexion angle increases^36^. The increased stiffness observed in the muscle-tendon-fascia was expected and might be explained by the stretching effect. This phenomenon seems to be physiologically plausible since anatomical characteristics indicate that the muscle-tendon-fascia unit becomes significantly stretched with an increasing ankle angle. In addition, the relatively higher stiffness of the AT can be explained by fascia or pulleys. The AT has a relatively short slack length, so it can be stretched before the muscle and its fascia are stretched^33^. Furthermore, the present study showed that only the stiffness of the PF-pro increased with increasing ankle dorsiflexion. This result explains why plantar fasciitis tends to occur at the proximal end of the PF. Changes in PF stiffness can affect the regulation of heel motion and control as well as joint stability. In theory, an increase in PF stiffness can cause a decreases in the capacity for PF to resist external loading and PF flexibility. At this time, if excessive and repetitive tensile forces are imposed onto the proximal end of the PF, microtrauma can develop, which may cause plantar fasciitis^8,19,34,36^. In summary, a warm-up (e.g., stretching) is necessary before individuals perform functional activities such as running and jumping. In this way, the muscle-tendon-fascia can be activated first, thereby reducing the risk of injury caused by large explosive forces or external forces during exercise.

### Effects of ankle and knee angle on the stiffness of the MG, LG and SOL

We report new findings for the MG and LG. When the knee was fully extended, the increase in stiffness of the MG and LG during passive ankle dorsiflexion and the percentage change in the MG were larger than those of the LG, especially at DF25°. This result means that passive movement of the ankle joint with the knee fully extended has the greatest effect on the MG. This result can be explained by anatomical factors. First, not only were the cross-sectional area (CSA) and volume of the MG larger than those of the LG^37^, but the MG was also longer than the LG, allowing it to extend more distally in the calf^9^. Second, the MG and LG have the same function in plantar flexion, but they have different contribution levels because the MG provides more than 70% of the force^3^. Third, the twisted structure of the AT is formed by MG tendons, LG tendons and SOL tendons. These muscle tendons rotate as they descend but do not run parallel to each other^4^. The torsion on the LG is significantly larger than that on the MG, and the rotation angle of the MG (28.17 ± 15.15°) is approximately one-fifth of that of the LG (135.98 ± 33.58°)^2^. Fourth, the smallest component of the AT insertion into the calcaneal bone is the LG (14.4 mm), and the largest is the MG (28.3 mm). The mean width of the footprint of the MG is approximately 2 times that of the LG^5^. Moreover, a previous study showed that the slack angle decreases significantly in the following order: LG > MG > AT^37^. Therefore, given all of the above factors, it is most likely that tension created during passive ankle dorsiflexion acts on the MG.

We also found that regardless of whether the knee was fully extended or flexed to 90°, the stiffness of the SOL was higher than those of the MG and LG, which may be related to the locations at which the measurements were taken and interactions between contractile and connective tissue elements^38^. In our study, SOL stiffness was measured at the distal region of the SOL, which is near the musculotendinous junction and is exposed to higher stresses than other regions of the SOL, which may result in increased stiffness^39^. In addition, the Cohen’s d and percentage change in the SOL were smaller than those in the MG and LG, likely because the MG and LG, along with the plantaris, cross the knee joint, while the origin of the SOL is located below the knee. Therefore, when the knee was fully extended, the MG and LG were stretched during passive ankle dorsiflexion. The extent that the SOL was stretched was smaller than those of the MG and LG. Additionally, the SOL is a postural muscle that is composed mainly of slow-twitch type I fibers, while the MG and LG consist mainly of fast-twitch fibers. In summary, regardless of the knee angle, the changes in SOL stiffness were smaller than those in the MG and LG.

We found that when the knee was fully extended, the increase in stiffness at PF50° was larger in the LG than in the MG and that no significant difference was observed at 0°, while the stiffness was higher in the MG than in the LG at DF25°. However, the same relationship did not hold when the knee was flexed to 90°. This result is consistent with that of Huang *et al*.^28^, who used MyotonPRO for their assessment. Hirata *et al*.^35^ also found that the stiffness of the MG was higher than that of the LG at DF25°. Moreover, Akagi and Takahashi^40^ found that the stiffness in the LG (33.5 ± 6.3 kPa) was higher than that in the MG (27.6 ± 7.3 kPa) at 30° of plantar flexion. Moreover, a previous study showed that the slack angle decreases significantly in the following order: LG > MG > AT^35^. Thus, stiffness in the MG might be decrease to a greater extent during plantar flexion than during dorsiflexion^4^. In other words, when the knee is fully extended, the slack angle of the LG is more dorsiflexed than that of the MG, and the slack angle of the MG is more plantar-flexed than that of the LG. This relationship may help explain why the MG is stiffer than the LG at DF25°. Muscle fiber type is another factor that may affect muscle stiffness because the stiffness of type I muscle fibers is higher than that of type II muscle fibers^41,42^. In addition, perimysium and endomysium can affect muscle stiffness^43^. Considering the above factors, it is reasonable that the stiffness in the LG was higher than that in the MG at PF50°, whereas that in the MG was higher than that in the LG at DF25°. According to the results of the present study, we speculate that the probability of MG injury is higher than that of LG injury. Moreover, we also suggest relaxing stiff muscles, such as the MG, after exercise. Additional studies need to be conducted in the future to confirm this hypothesis and suggestion.

### Effects of ankle and knee angle on AT stiffness

Regardless of the knee angle, stiffness decreased from AT3cm to AT0cm to AT6cm at PF50° and 0°, while the stiffness decreased from AT0cm to AT3cm to AT6cm at DF25°. The main function of the AT is to act as a mechanical buffer to absorb energy during plantar flexion of the ankle and prevent soft tissue injury resulting from a sudden increase in external or internal forces^44^. In addition, findings from previous studies have shown that the AT had an inherently high stiffness. This result may be explained by the AT having a relatively small CSA yet experiencing inhomogeneous mechanical loading during daily activities, making the tendon susceptible to microtears and the type of gene expression that promotes type I collagen synthesis^45^. The AT has high viscoelasticity and mechanical strength, enabling it to respond to altered mechanical loading conditions^46^. Thus, if the AT is too stiff, it cannot absorb sufficient energy, and thus, there is an increased risk of injury. In addition, stiff tendons cannot transmit forces to neighboring tendons or muscles quickly and effectively and thus are more likely to be injured^47^. According to the results of this study, AT stiffness increases with increasing ankle dorsiflexion; when the stiffness is high, the risk of AT injuries or tears increases with increasing ankle dorsiflexion angles. According to the distribution of blood vessels, AT3cm is one of the areas with the least perfusion^48^. As mentioned above, combined with the lack of vascular perfusion and the high sensitivity of the AT3cm during ankle dorsiflexion, we speculate that the injury risk at AT3cm is high and that injuries in this area heal poorly. In addition, the stiffness decreased from AT0cm to AT3cm to AT6cm at DF25°. The reason for this result was that at ankle angles approaching the maximum range of motion, the magnitude of myotendinous junction displacement has reached a maximum, and the relative magnitude of tendon elongation has reached a minimum^32,38,49,50^. Therefore, more energy must be absorbed by the AT, and AT0cm is close to the calcaneus, so it incurs the largest component of the passive tensile force. Therefore, AT0cm has the highest stiffness at DF25° compared with other angles.

Interestingly, we found that the angles of flexion and extension of the knee and ankle joints have a large effect on the stiffness of the MG, LG and AT; however, the PF and SOL are only greatly affected by the ankle joint angle. This seems to be physiologically plausible since anatomical studies indicate that the MG, LG and AT, along with the plantaris, cross the knee joint, while the origin of the PF is located below the calcaneal tuberosity, and the origin of the SOL is located below the knee. Therefore, when the knee is fully extended, the MG, LG and AT are stretched, as are the SOL and PF, which do not act on the knee joint.

As the results from the present study apply only to young participants who are healthy and free of injury or disease, additional studies need to be conducted in the future to quantify the stiffness distribution in patients who present with muscle contractures or tendon/PF injuries due to musculoskeletal or neuronal conditions. At present, the locations of muscle contractures or tendon/PF injuries and PF stiffness are difficult to quantify. Clinically, manual examinations only provide subjective and overall outcomes and do not provide specific values for each region. The design and protocol used in the present study may be applied in studies in patients to identify the locations that actually incur injuries to better prescribe targeted treatments, such as stretching, myofascial release and shock wave therapy. Moreover, if similar results are confirmed among patients, SWE measurements should be used for treatment efficiency and follow-up assessment purposes.

### Limitations

The SWE technique can be used to identify biomechanical properties of muscles and tendons, but several technical parameters that influence the measurements still need to be addressed. First, it should be noted that the SWE technique is based on the assumption that the tissues being imaged are homogenous, isotropic and purely elastic. However, tissues do not always meet these criteria, and further consideration of the effectiveness of the algorithm is necessary for the use of the SWE technique in muscles, tendons and fascial stiffness assessments. Therefore, the angle between the orientation of the fibers and the probe influences the accuracy of the measurements. In this study, the midpoint of the transducer was placed perpendicular to the muscle/tendon on the skin’s surface, where we had previously made a mark, with light pressure, ensuring that the transducer was aligned with the longitudinal axis of the muscles/tendons to minimize the anisotropic effect on the measurement results. In addition, given that the muscles, tendons and fascia are not homogenous, isotropic or purely elastic, the present study only reported the Young’s modulus. In future studies, we will use two units of measurement (m/s and kPa). Second, SWE cannot be used to measure deep tissues. The stiffness of the SOL belly could not be assessed because its location was too deep. Therefore, the distal portion of the SOL was used for the measurements. Another limitation of our study is that EMG was not used to monitor muscle activity and ensure that the muscles did not contract during the tests. However, all volunteers were asked to remain relaxed, and no signs of muscle contractions were visible on the B-mode images. Therefore, we are confident that all volunteers remained in a relaxed state and followed the verbal instructions. The architecture and size of the muscle-tendon-fascia were not assessed in the present study. The force-generating capacity of muscles depends on their CSA and pennation angle. Although the shear modulus may not be affected by the anatomical CSA and pennation angle of the muscle, future studies still need to consider these parameters in patients^51^. Only male subjects were recruited in this study. In our pilot experiment, we found that the myofascial thickness of females was less than 1 mm, while males was larger than 1 mm. Therefore, we only recruited males in this study, and the results of the present study can not be generalized to female subjects. We need to develop more accurate algorithms and more diversified Q-boxes to meet the needs of future studies.

## Conclusion

Ankle dorsiflexion induces nonhomogeneous changes in tensile stress between the main lower limb muscles and within the tendon/PF. This study provides a valuable reference for studies in which individual muscle/tendon/PF contributions are estimated.

## Data Availability

All data included in this study are available upon request by contact with the corresponding author.

## References

[1] Fahlstrom M (2003). Chronic Achilles tendon pain treated with eccentric calf-muscle training. Knee Surg. Sports Traumatol. Arthrosc..

[2] Pękala PA (2017). The twisted structure of the Achilles tendon unraveled: A detailed quantitative and qualitative anatomical investigation. Scand. J. Med. Sci. Sports..

[3] Dayton P (2017). Anatomic, Vascular, and Mechanical Overview of the Achilles Tendon. Clin. Podiatr. Med. Surg..

[4] Edama M (2015). The twisted structure of the human Achilles tendon. Scand. J. Med. Sci. Sports..

[5] Ballal MS, Walker CR, Molloy AP (2014). The anatomical footprint of the Achilles tendon: a cadaveric study. Bone Jt. J..

[6] O’Brien M (2005). The anatomy of the Achilles tendon. Foot Ankle Clin..

[7] Romero-Morales C (2019). Comparison of the sonographic features of the Achilles Tendon complex in patients with and without achilles tendinopathy: A case-control study. Phys. Ther. Sport..

[8] Putz FJ, Hautmann MG, Banas MC, Jung EM (2017). Investigation of the acute plantar fasciitis with contrast-enhanced ultrasound and shear wave elastography - first results. ClinHemorheolMicrocirc..

[9] Somers, K; *et al*. Acute Effects of Gastrocnemius/Soleus Self-Myofascial Release Versus Dynamic Stretching on Closed-Chain Dorsiflexion.[J]. *J Sport Rehabil*. 1-7 (2019). 10.1123/jsr.2018-0199 30747565

[10] Magnan B (2014). The pathogenesis of Achilles tendinopathy: a systematic review.[J]. Foot Ankle Surg..

[11] Schneider HP (2018). American College of Foot and Ankle Surgeons Clinical Consensus Statement: Diagnosis and Treatment of Adult Acquired Infracalcaneal Heel Pain.[J]. J. Foot Ankle Surg..

[12] Attias M (2016). Effects of contracture on gait kinematics: a systematic review. Clin. Biomech..

[13] Schneider S (2015). Feasibility of monitoring muscle health in microgravity environments using Myoton technology. Med. Biol. Eng. Comput..

[14] Green M (2013). Measuring anisotropic muscle stiffness propertiesusing elastography. NMR Biomed..

[15] Orner S (2018). Quant. tissue parameters Achilles tendon plantar fascia healthy participants using. a handheld myotonometer.[J].J Bodyw. Mov. Ther..

[16] Creze M (2018). Shear wave sonoelastography of skeletal muscle: basic principles, biomechanical concepts, clinical applications, and future perspectives. Skelet. Radiol..

[17] Zhou J (2019). Regional Elastic Properties of the Achilles Tendon Is Heterogeneously Influenced by Individual Muscle of the Gastrocnemius. Appl. Bionics Biomech..

[18] Eby SF (2013). Validation of shear wave elastography in skeletal muscle. J. Biomechanics..

[19] Wu CH (2012). Can sonoelastography detect plantar fasciitis earlier than traditional B-mode ultrasonography?. Am. J. Phys. Med. Rehabil..

[20] Wu CH, Wang TG (2012). Measurement of muscle stiffness in children with spastic cerebral palsy. Radiology..

[21] Wu CH (2017). Evaluation of Post-Stroke Spastic Muscle Stiffness Using Shear Wave Ultrasound Elastography. Ultrasound Med. Biol..

[22] Fu S, Cui L, He X, Sun Y (2016). Elastic Characteristics of the Normal Achilles Tendon Assessed by Virtual Touch Imaging Quantification Shear Wave Elastography. J. Ultrasound Med..

[23] Wu CH (2011). Sonoelastography of the plantar fascia. Radiology..

[24] Payne C (2018). Reproducibility of shear wave elastography measuresof the Achilles tendon. Skelet. Radiol..

[25] Mahieu NN (2008). Effect of eccentric training on the plantar flexor muscle-tendon tissue properties. Med. Sci. Sports Exerc..

[26] Haen TX (2017). Shear waves elastography for assessment of human Achilles tendon’s biomechanical properties: an experimental study. J. Mech. Behav. Biomed. Mater..

[27] Finni T (2017). Effects of muscle activation on shear between human soleus and gastrocnemius muscles. Scand. J. Med. Sci. Sports..

[28] Huang J (2018). Assessment of Passive Stiffness of Medial and Lateral Heads of Gastrocnemius Muscle, Achilles Tendon, and Plantar Fascia at Different Ankle and Knee Positions Using the MyotonPRO. Med. Sci. Monit..

[29] Bouvier T (2017). Acute effects of static stretching on muscle-tendon mechanics of quadriceps and plantar flexor muscles. Eur. J. Appl. Physiol..

[30] Cohen J. Statistical power analysis for the behavioral sciences 2nd ed. Erlbaum Associates, Hillsdale (1998).

[31] Maïsetti O (2012). Characterization of passive elastic properties of the human medial gastrocnemius muscle belly using supersonic shear imaging. J. Biomech..

[32] Hug F (2013). Slack length of gastrocnemius medialis and Achilles tendon occurs at different ankle angles. J. Biomech..

[33] Le Sant G (2017). Stiffness mapping of lower leg muscles during passive dorsiflexion. J. Anat..

[34] Carlson RE, Fleming LL, Hutton WC (2000). The biomechanical relationship between the tendoachilles, plantar fascia and metatarsophalangeal joint dorsiflexion angle. Foot Ankle Int..

[35] Hirata K (2016). Muscle-specific acute changes in passive stiffness of human triceps surae after stretching. Eur. J. Appl. Physiol..

[36] Cheng HY (2008). Finite element analysis of plantar fascia under stretch-the relative contribution of windlass mechanism and Achilles tendon force. J. Biomech..

[37] Toumi H (2016). Implications of the calf musculature and Achilles tendon architectures for understanding the site of injury. J. Biomech..

[38] MacDonald D (2016). Reliability of Abdominal Muscle Stiffness Measured Using Elastography during Trunk Rehabilitation Exercises. Ultrasound Med. Biol..

[39] Kjær M (2004). Role of extracellular matrix in adaptation of tendon and skeletal muscle to mechanical loading. Physiol. Rev..

[40] Akagi R, Takahashi H (2013). Acute effect of static stretching on hardness of the gastrocnemius muscle. Med. Sci. Sport. Exer..

[41] Mutungi, G., Ranatunga, K. The viscous, viscoelastic and elastic characteristics of resting fast and slow mammalian (rat) muscle fibres. *J. Physiol*. **496**(3), 827–836 (1996). 10.1113/jphysiol.1996.sp021730 PMC1160867 8930847

[42] Mutungi, G., Ranatunga, K. Temperature‐dependent changes in the viscoelasticity of intact resting mammalian (rat) fast‐and slow‐twitch muscle fibres. *J. Physiol*. **508**(1), 253–265 (1998). 10.1111/j.1469-7793.1998.253br.x PMC2230871 9490847

[43] Gajdosik RL (2001). Passive extensibility of skeletal muscle:review of the literature with clinical implications. Clin. Biomech..

[44] Zhang L (2016). Evaluation of elastic stiffness in healing achilles tendon after surgical repair of a tendon rupture using *in vivo* ultrasound shear wave elastography. Med. Sci. Monit..

[45] Chiu TC (2016). An Investigation of the Immediate Effect of Static Stretching on the Morphology and Stiffness of Achilles Tendon inDominant and Non-Dominant Legs. PLoS One..

[46] Wang JH, Guo Q, Li B (2012). Tendon biomechanics and mechanobiology–a minireview of basic concepts and recent advancements. J. Hand Ther..

[47] Butler RJ, Iii HPC, Davis MC (2003). Lower extremity stiffness: Implications for performance and injury. Clin. Biomech..

[48] Lagergren C, Lindholm A (1959). Vascular distribution in the Achilles tendon; An angiographic and microangiographic study. Acta Chir. Scand..

[49] Nakamura M, Ikezoe T, Takeno Y, Ichihashi N (2011). Acute and prolonged effect of static stretching on the passive stiffness of the human gastrocnemius muscle tendon unit *in vivo*. J. Orthop. Res..

[50] Nakamura, M., Ikezoe, T., Takeno, Y. & Ichihashi, N. Time course of changes in passive properties of the gastrocnemius muscle-tendon unit during 5 min of static stretching. *Man Ther*. 211-215 (2013). 10.1016/j.math.2012.09.010 23294911

[51] Miyamoto N (2015). Validity of measurement of shear modulus by ultrasound shear wave elastography in human pennate muscle. PLoS One..

